# Changes in land use alter soil quality and aggregate stability in the highlands of northern Ethiopia

**DOI:** 10.1038/s41598-017-14128-y

**Published:** 2017-10-19

**Authors:** Yoseph T. Delelegn, Witoon Purahong, Amila Blazevic, Birru Yitaferu, Tesfaye Wubet, Hans Göransson, Douglas L. Godbold

**Affiliations:** 1 BOKU–University of Natural Resources and Life Sciences, Institute of Forest Ecology, Peter-Jordan-Straße 82, 1190 Vienna, Austria; 2 UFZ-Helmholtz Centre for Environmental Research, Department of Soil Ecology, Theodor-Lieser-Str.4, D-06120 Halle (Saale), Germany; 3 ARARI–Amhara Regional Agricultural Research Institute, P. O. Box 527 Bahar Dar, Ethiopia

## Abstract

Land use change alters biodiversity and soil quality and thus affects ecosystem functions. This study investigated the effects of changes in land use on major soil quality indicators. Soil samples were taken from a depth of 0–10 cm (top soil) under four major land uses (cropland, grassland, area exclosure, eucalyptus plantation) with similar land use change histories for analysis, and soil from a nearby natural forest was used as a reference. Land use change from natural forest to cropland and grassland significantly decreased major soil quality indicators such as soil organic C (SOC), total soil N (TSN), molybdate-reactive bicarbonate-extractable P, and arbuscular mycorrhizal fungi (AMF) spore density, but compared to the cropland, change to area exclosure and eucalyptus plantation significantly improved SOC, TSN and soil aggregate stability (SAS). In addition, we assessed the correlation among indicators and found that SOC, TSN and SAS significantly correlate with many other soil quality indicators. The study highlights that the conversion of natural forest to cropland results in decline of soil quality and aggregate stability. However, compared to cropland, application of area exclosure and afforestation on degraded lands restores soil quality and aggregate stability.

## Introduction

Changes in land use significantly alter biodiversity and ecosystem functioning across different biomes and continents^1,2^, but the effects of such change are often mediated by variations in the physical, chemical and biological qualities of soils^3^. Land use-mediated soil degradation is pronounced in the tropics^4,5^, where the combination of climate and poor soil management accelerates decomposition and soil nutrient loss. Africa in general and East Africa in particular are characterized by the accelerated deterioration of soil quality and stability^6–8^, which has affected agricultural productivity, food security and the overall resilience of the socio-ecological system^9^. Proper land and soil management is needed to maintain soil quality and aggregate stability.

Understanding land use history is essential to comprehend the magnitude and trend of changes in soil quality^10^, and the highlands of northern Ethiopia, where most of the natural forests have been converted to other land use types, provide an interesting context to investigate these aspects. Considerable areas of natural forests in the highlands have been converted to croplands and grasslands^11,12^. This includes the study location, which was once covered by natural forest, but following human settlement, large tracts of the natural landscape has been slowly cleared for agricultural activities. As the population increased, eucalyptus plantations were introduced to the area in the 1980s as sources of fuelwood to buffer the remaining natural forests. The eucalyptus trees were planted on lands that had been converted from natural forest and became marginalized due to overgrazing and poor land management. The agricultural system in this region of Ethiopia is characterized by low-input and low-output production system. Soil management practices are poor, and productivity is maintained through extensification rather than intensification^9^.

Additionally, eucalyptus plantation is a major land use that was established by the Ethiopian government to address the growing demand for wood products^13^, and the expansion of this land use has significantly contributed to an increase in total forest plantation cover from an estimated 190,000 ha in 1990 to approximately 972,000 ha in 2010^14^. Despite their economic importance, there are ecological controversies surrounding eucalyptus plantations including their effect on soil quality^15^. Some studies have reported negative impacts of eucalyptus plantations on soil quality compared to conventional cropland management^15,16^, whereas others have demonstrated positive ecological effects of plantation trees as nurse trees that stimulate the regeneration of native woody plants^17^. Other studies have also shown an increase in soil organic matter following afforestation of degraded lands with eucalyptus^13,18^, but there is still little knowledge about the effect of planting eucalyptus on other soil parameters.

Soil quality and stability under different land uses can be measured using indicators that interact synergistically. According to Karlen *et al*.^19^, biophysical and chemical indicators can be useful for monitoring soil quality, but it is often the chemical properties of the soil rather than the combination of physical and biological properties that are used to evaluate soil quality^19,20^.

Soil quality is the capacity of a soil to function and promote plant and animal productivity, and maintain or enhance water and air quality^21^. Maintaining and promoting soil quality is, therefore, a fundamental requirement to ensure ecosystem sustainability. Soil aggregate stability is the capacity of soil to resist mechanical disruptions that may leads to soil erosion. It is one of the key physical soil properties affecting soil pore space, soil structure and root penetration and thus it is used as an important physical indicator of soil quality^22,23^. Some studies suggest that soil aggregate stability can be used as a simple proxy for factors such as organic matter turnover, soil biochemical activities, soil erodibility, soil hydrology processes, and the resilience of the soil to drought as well as an indicator of ecosystem sustainability^22,24,25^. In ecosystem restoration studies, it is also considered as a promising indicator of the restoration status in eroded ecosystems, where the land use change is associated with change in plant community composition^26^.

Soil aggregate stability is often regulated by factors such as soil organic matter, microbial biomass, iron and aluminum oxides, and other clay minerals^27,28^. Additionally, biological properties including microbes (e.g., arbuscular mycorrhizal fungi - AMF) and their biological activities (i.e., soil enzyme activities) are key indicators of soil quality and stability. The accumulation of AMF hyphal biomass and hyphal exudates has been shown to be important for the accumulation of soil organic carbon and soil structural stability^28–30^. The functional role of AMF is strongly linked to the composition of fungal cell walls, such as the polymers of melanin and chitin that are resistant to decomposition^31^. Moreover, AMF plays a role in promoting the formation of macro-aggregates through the biophysical functions of gluing and the hyphal entanglement of micro-aggregates^32^. There is growing evidence that soil enzyme activities related to the biocycles of elements (C, N, P and S) may hold potential as early and sensitive indicators of soil ecological stress or restoration^33–35^. Soil enzymes come from both symbiotic and non-symbiotic microbial communities that are driven by the need for and availability of C and other soil nutrients. Therefore, potential enzyme activity can be used to evaluate the impact of land use changes on the turnover of soil organic carbon and nutrient mineralization^25,36^.

Although there have been some attempts to assess the impacts of land use changes on soil quality in the highlands of Ethiopia, none have integrated parameters from the three key soil quality indicators (i.e., chemical, physical and biological). In this study, we investigated five land uses, namely, cropland, grassland, area exclosure, eucalyptus plantation and natural forest, to investigate the effects of land use on the three soil quality indicators; the natural forest was used as a reference to measure changes and make comparisons among land uses. We investigated a number of chemical, biological and physical attributes such as pH, SOC, TSN, AMF spore density, the enzyme β-glucosidase to represent C acquisition, chitinase and protease to represent N acquisition, phosphatase to represent P acquisition^37^, and SAS. We aimed to (i) investigate the effect of different land uses (natural forest, cropland, grassland, and degraded land converted to area exclosure or planted with Eucalyptus) on soil quality and aggregate stability and (ii) to assess the relative efficiency of soil quality indicators for land management and monitoring purposes. We hypothesized that (i) changes in land use alter major soil quality indicators and that (ii) cropland as a land use leads to the deterioration of all soil quality parameters, whereas the recovery of natural vegetation and afforestation supports the restoration of soil quality and stability. By knowing how the different land uses affect the soil quality farmers can better plan their activities to maintain soil quality and thereby maintain long term productivity.

## Results

### Soil pH, carbon, nitrogen and phosphorus

**Figure 1:**
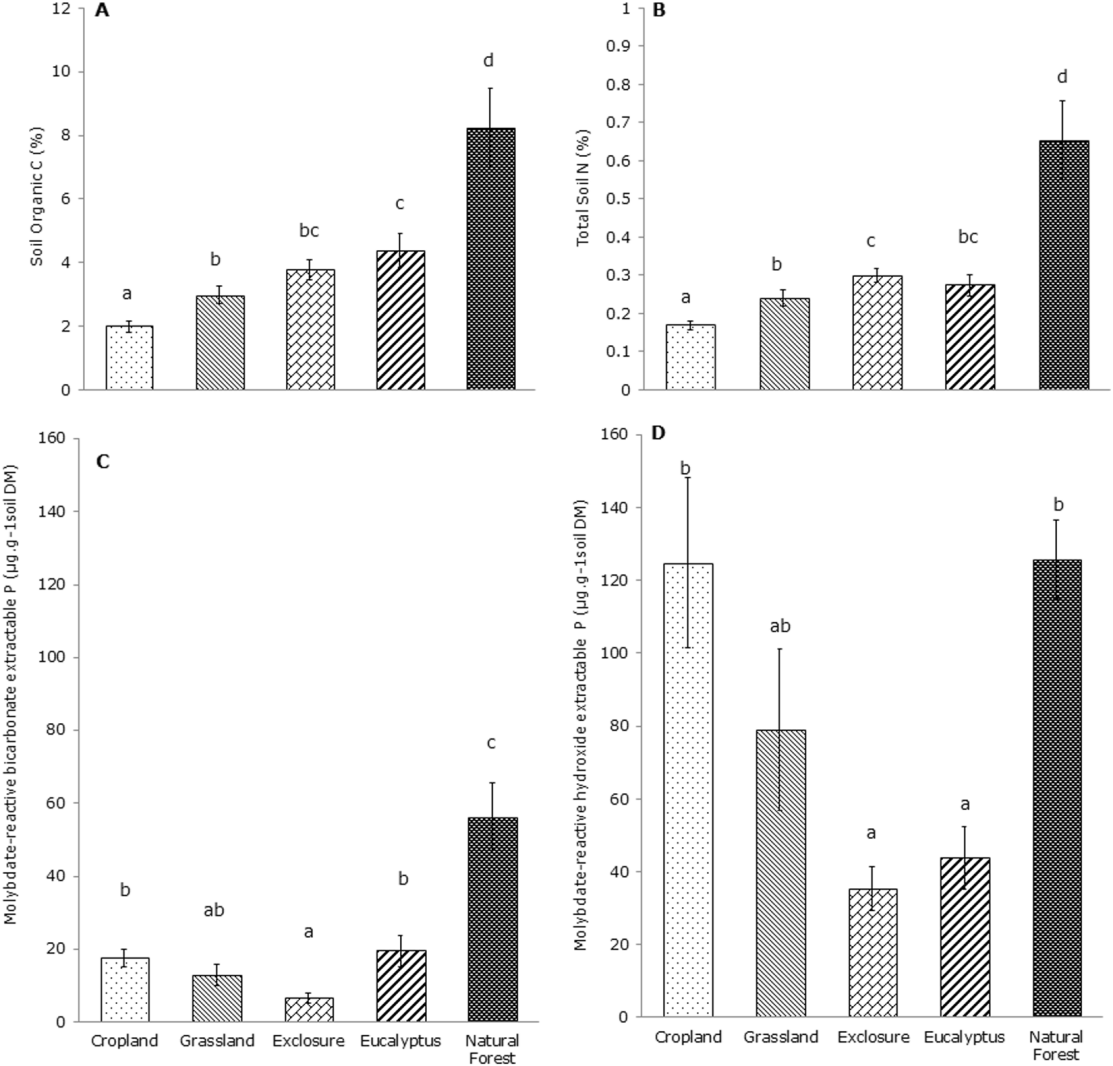
Soil organic C, TSN and extractable P fractions (mean ± SE) in the soils of five different land use types in the highlands of northern Ethiopia. Different letters indicate statistically significant differences (*P* < 0.05) between land uses according to one-way ANOVA and Tukey’s honest significance test (molybdate-reactive bicarbonate P fraction, molybdate-reactive hydroxide P fraction) or a Kruskal–Wallis test and Mann–Whitney U test (SOC and TSN).

The soil pH of the five land uses ranged between 6.37 and 6.89, and only that of the soil from the eucalyptus stand was significantly lower than that of the other land use types (Table 1). The natural forest soil had substantially higher SOC and TSN than the soils of the other studied land uses, whereas the cropland soil had the lowest SOC and TSN (Fig. 1A and B). The SOC from the area exclosure was not statistically significantly different from that of the grassland and eucalyptus plantation (*P* = 0.05; *P* = 0.31, respectively), but the SOC in the eucalyptus plantation was significantly higher than that in the cropland and grassland (*P* < 0.01; *P* < 0.05, respectively) (Fig. 1A). The TSN measured in the eucalyptus plantation was not significantly different from the TSN in the grassland and exclosure (*P* = 0.31; *P* = 0.25, respectively), but the TSN in the exclosure was significantly higher than that determined for the cropland and grassland (*P* < 0.01; *P* < 0.05, respectively) (Fig. 1B).

**Table 1:** Soil pH (0- to 10-cm soil depth) of soils from different land use types in Ambo Ber, Ethiopia. Values followed by different letters are significantly different (P < 0.05).

Land use types	Soil pH (±SE)
Cropland	6.61 ± 0.05ab
Grassland	6.74 ± 0.04b
Exclosure	6.89 ± 0.09b
Eucalyptus plantation	6.37 ± 0.10a
Natural forest	6.86 ± 0.10b

The molybdate-reactive bicarbonate-extractable P fraction (bicarbonate-extractable P) in the soil of the natural forest was significantly higher than that in the soils of the other land uses (Fig. 1C), and that in the exclosure soil was significantly lower than in the cropland, eucalyptus and natural forest soils but not significantly different from that of the grassland soil (*P* > 0.05) (Fig. 1C). There was no statistically significant difference in the bicarbonate-extractable P content among the cropland, grassland and eucalyptus plantation soils (*P* > 0.05). The molybdate-reactive hydroxide-extractable P fraction (hydroxide-extractable P) measured in the cropland and grassland soils was not significantly different from that of the natural forest (*P* > 0.05) (Fig. 1D), and that of the exclosure and eucalyptus plantation soils was significantly lower than in the natural forest soil and cropland (*P* < 0.01) (Fig. 1D).

**Figure 2:**
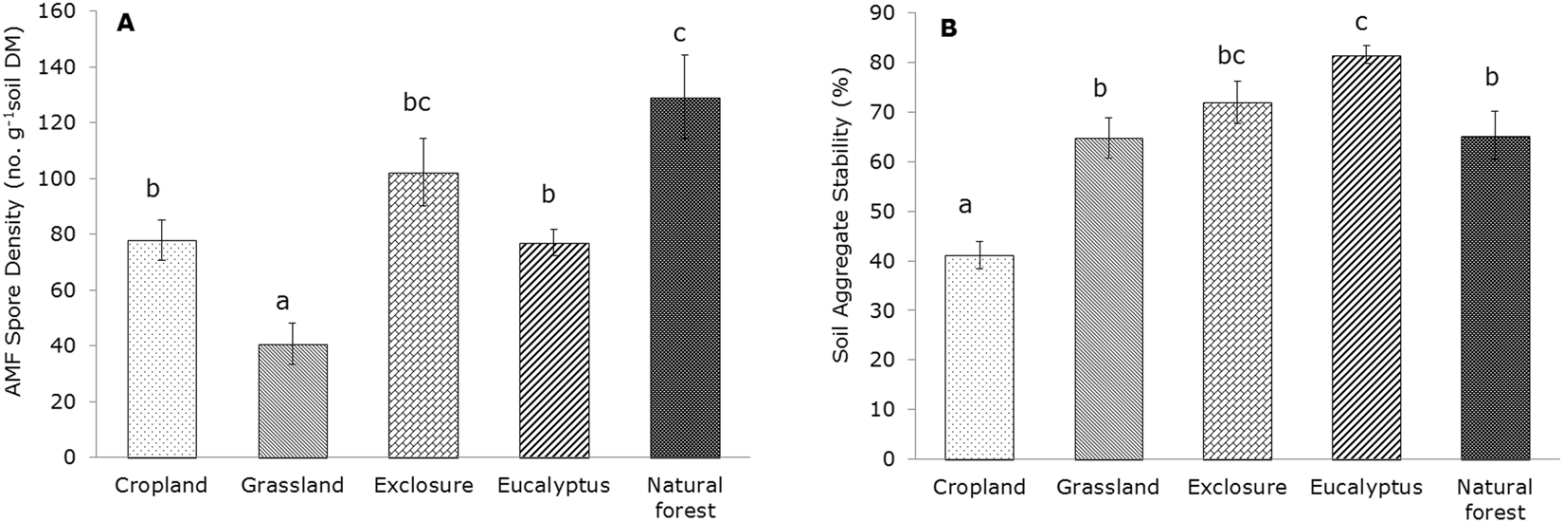
AMF spore density and percent wet soil aggregate stability (mean ± SE) in the soils of five different land use types in the highlands of northern Ethiopia. Different letters indicate significant differences (*P* < 0.05) between land uses according to one-way ANOVA and Tukey’s honest significance test for wet soil aggregate stability and a Kruskal–Wallis test and Mann–Whitney U test for spore density.

The natural forest soil had a significantly higher AMF spore density (AMF-SD) per gram of dry soil (129 spore g^−1^) than the soils from the other land uses, except area exclosure (102 spore g^−1^) in which the AMF-SD was significantly higher than in the grassland soil (41 spore g^−1^) (Fig. 2A). There were no significant differences among the soils sampled from the cropland (78 spore g^−1^), exclosure (102 spore g^−1^) and eucalyptus plantation (77 spore g^−1^) (Fig. 2A). The AMF-SD was significantly correlated with the SOC and TSN (*ρ* = 0.45, *P* < 0.01; *ρ* = 0.44, *P* < 0.01, respectively) (Table 2), but there was a negative correlation between the AMF-SD and *β*-glucosidase (*ρ* = −0.44, *P* < 0.01) (Table 2).

**Table 2:** Spearman’s rank correlations between soil aggregate stability and AMF spore density and the soil quality indicators of soil pH, molybdate-reactive bicarbonate P fraction, molybdate-reactive hydroxide P fraction, soil organic C and total soil N. Significant correlations (*P* < 0.05) are indicated in bold.

Soil quality indicators	Aggregate stability		AMF spore density	
	*ρ*	*P*	*ρ*	*P*
Aggregate stability	n.d.	n.d.	0.02	0.879
AMF Spore density	0.02	0.879	n.d.	n.d.
Soil pH	−0.09	0.541	0.20	0.186
Molybdate-reactive bicarbonate P	−0.17	0.277	0.20	0.181
Molybdate-reactive hydroxide P	−**0.43**	**0.005**	0.12	0.446
Soil organic C	**0.46**	**0.003**	**0.45**	**0.002**
Total soil N	**0.34**	**0.034**	**0.44**	**0.003**
*β*-glucosidase	**0.35**	**0.020**	−**0.44**	**0.002**
Chitinase	**0.42**	**0.006**	−0.25	0.093

### Soil aggregate stability

The SAS (Fig. 2B) was statistically significantly higher in the eucalyptus plantation (82%) than in the other land use types, except in the exclosure (72%), and the lowest value was found in the cropland soil (41%). The SAS of the natural forest, area exclosure and grassland were not statistically significantly different (*P* > 0.05) (Fig. 2B). The Spearman’s rank correlation analysis showed significantly positive relationships between SAS and SOC, SAS and TSN, SAS and *β*-glucosidase and SAS and chitinase (*ρ* = 0.46, *P* < 0.01; *ρ* = 0.34, *P* < 0.05; *ρ* = 0.35, *P* < 0.05; *ρ* = 0.42, *P* < 0.01, respectively) (Table 2), whereas a negative correlation was found between SAS and hydroxide-extractable P (*ρ* = −0.44, *P* < 0.01) (Table 2). Even though both aggregate stability and spore density were positively correlated with SOC, there was weak direct correlation between the two (*ρ* = 0.02, *P* = 0.88) (Table 2).

### Potential soil enzyme activities across the land uses

**Figure 3:**
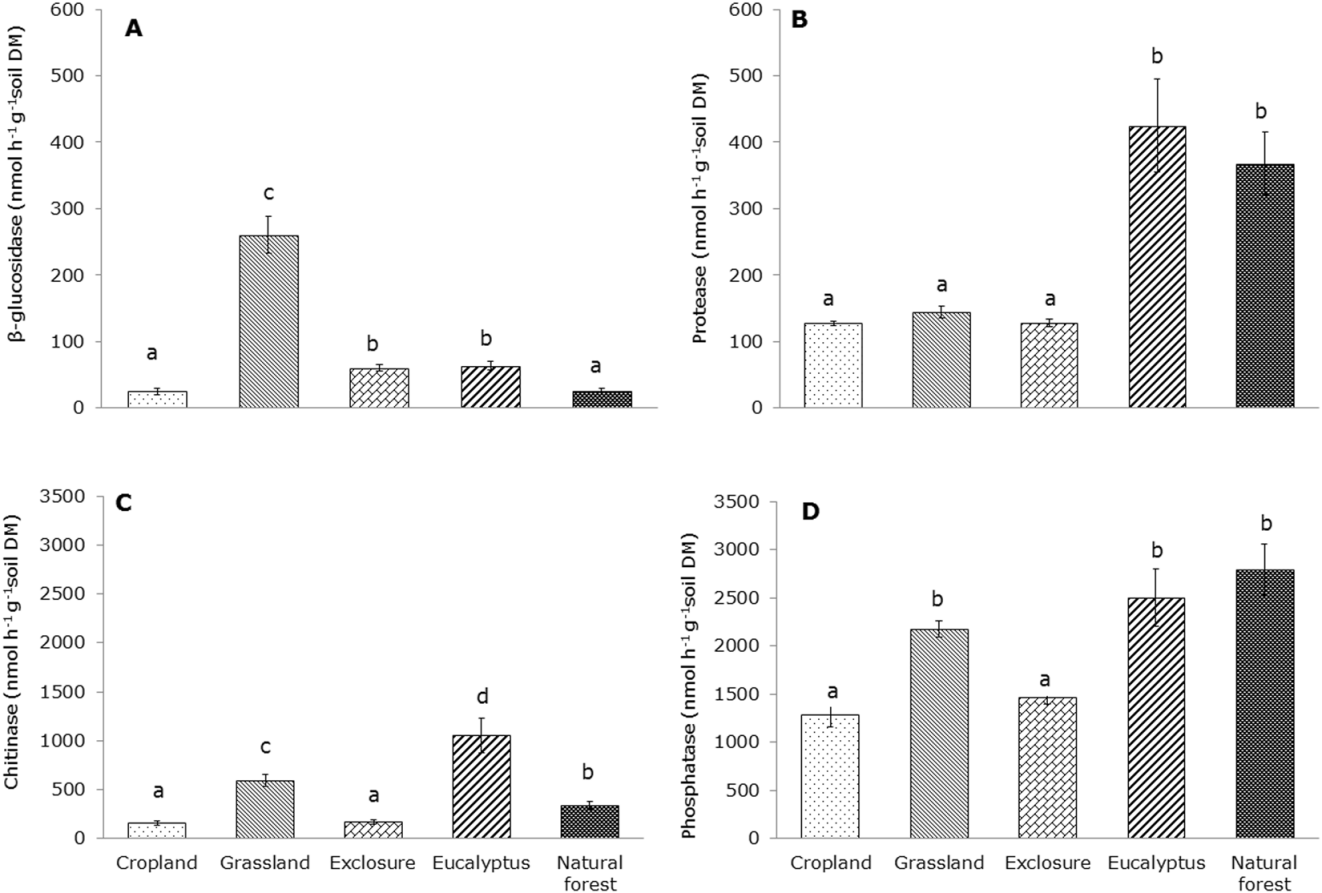
Soil enzyme activities (mean ± SE) in the soils of five different land uses in the highlands of northern Ethiopia. Different letters indicate significant differences (*P* < 0.05) between different land uses according to a Kruskal–Wallis test and Mann–Whitney U test.

The grassland soil had significantly higher *β*-glucosidase (260 nmol h^−1^ g^−1^ soil DM) than the soils of the other land uses (Fig. 3A), and the cropland and natural forest soils had the lowest *β*-glucosidase contents, 24 nmol h^−1^ g^−1^ soil DM for both (Fig. 3A). The soils in the eucalyptus plantation and the natural forest showed significantly higher protease activities than the other land uses, and there no significant difference was detected between the cropland, grassland and exclosure soils (*P* > 0.05) (Fig. 3B). The protease activity was positively correlated with bicarbonate-extractable P, SOC and TSN (*ρ* = 0.40, *P* < 0.01; *ρ* = 0.40, *P* < 0.01; *ρ* = 0.39, *P* < 0.01, respectively) (Table 3). The chitinase activity was significantly higher in the soils of the eucalyptus plantation (ca. 1,051 nmol h^−1^ g^−1^ soil DM) than in the soils of the other land use types, and the lowest activity was observed in the cropland and exclosure soils (ca. 158 and 174 nmol h^−1^ g^−1^ soil DM, respectively) (Fig. 3C). The chitinase activity was negatively correlated with soil pH (*ρ* = −0.32, *P* < 0.05), but a significantly positive correlation was found with SAS (*ρ* = 0.42, *P* < 0.01) (Table 3). The acid phosphatase activity was substantially higher in the grassland, eucalyptus plantation and natural forest soils than in the cropland and exclosure soils (Fig. 3A), and it was positively correlated with the bicarbonate-extractable P fraction, SOC, and TSN (*ρ* = 0.38, *P* < 0.05; *ρ* = 0.48, *P* < 0.01; *ρ* = 0.47, *P* < 0.01, respectively) (Table 3).

**Table 3:** Spearman’s rank correlations between enzyme activities and spore density, aggregate stability, soil pH molybdate-reactive bicarbonate P fraction, molybdate-reactive hydroxide P fraction, soil organic C, and total soil N. Significant correlations (*P* < 0.05) are indicated in bold.

Factor	*β*-glucosidase	Phosphatase	Chitinase	Protease
Spore density	−**0.44**	0.14	−0.25	0.07
Aggregate stability	**0.35**	0.12	**0.42**	0.29
Soil pH	0.00	−0.11	−**0.32**	−0.03
Molybdate-reactive bicarbonate P	−0.30	**0.38**	0.21	**0.40**
Molybdate-reactive hydroxide P	−**0.33**	0.20	−0.02	0.26
Soil organic C	0.03	**0.48**	0.27	**0.40**
Total soil N	0.03	**0.47**	0.13	**0.39**

**Figure 4:**
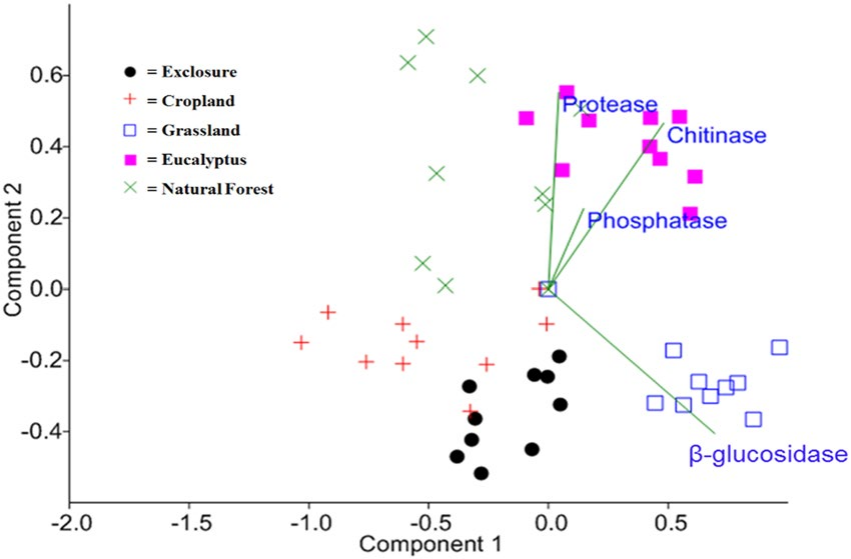
Overall patterns of enzyme activities across five different land uses calculated by principal component analysis (PCA with a biplot function). Two PCA components explain 90.2% of the variance in total enzyme activities. Significant deviations in enzyme activity patterns among different land uses were analyzed by analysis of similarity (ANOSIM). *P* values are Bonferroni corrected in all cases.

Chitinase, acid phosphatase and *β*-glucosidase correlated with PCA component-1 of the overall enzyme activity, which explained 60.2% of the overall variance, whereas only protease correlated with PCA component-2, which explained 30% of the overall variance (Fig. 4 and Table 4). The overall enzyme activity patterns were significantly different across the land use types (Fig. 4 and Table 5), and the analysis of similarity showed a clear separation of the activities between eucalyptus plantation and exclosure (*ρ* = 0.91, *P* < 0.01), eucalyptus plantation and cropland (*ρ* = 0.84, *P* < 0.05), and exclosure and grassland (*ρ* = 0.79, *P* < 0.01) (Table 5). However, some overlap of the overall enzyme activities was observed between natural forest and eucalyptus plantation (*ρ* = 0.38, *P* < 0.01) and exclosure and cropland (*ρ* = 0.33, *P* < 0.05) (Table 5).

**Table 4:** Spearman’s rank correlations between soil quality indicators (spore density, aggregate stability, soil pH, molybdate-reactive bicarbonate P fraction, molybdate-reactive hydroxide P fraction, soil organic C and total soil N) and the two main components of a principle component analysis (PCA) ordination calculated from four enzyme activities. Significant correlations (*P* < 0.05) are indicated in bold.

Soil quality indicators	PCA axis 1		PCA axis 2	
	*ρ*	*P*	*ρ*	*P*
AMF Spore density	−**0.38**	**0.006**	0.24	0.098
Aggregate stability	**0.40**	**0.005**	0.17	0.245
Soil pH	−0.17	0.263	−**0.30**	**0.049**
Molybdate-reactive bicarbonate P	−0.05	0.761	**0.54**	**0.001**
Molybdate-reactive hydroxide P	−0.20	0.206	**0.31**	**0.040**
Soil organic C	0.15	0.322	**0.30**	**0.045**
Total soil N	0.10	0.526	0.17	0.269

**Table 5:** Bray-Curtis analysis of similarity (ANOSIM) of the overall pattern of enzyme activities across the land use types (where *P* < 0.05 and an R-value between 0–0.299 indicates “no separation/overlap”; 0.300–0.749 indicates “different but with some overlap”; and >0.750 indicates “well separated” and is marked in bold).

Treatment	*ρ*	*P*
Overall	0.63	0.01
Natural forest vs. Eucalyptus plantation	0.38	0.01
Natural forest vs. Exclosure	0.68	0.01
Natural forest vs. Grassland	0.64	0.01
Natural forest vs. Cropland	0.51	0.01
Eucalyptus plantation vs. Exclosure	**0.91**	**0.01**
Eucalyptus plantation vs. Grassland	0.67	0.01
Eucalyptus plantation Vs. Cropland	**0.84**	**0.02**
Exclosure vs. Grassland	**0.79**	**0.01**
Exclosure vs. Cropland	0.33	0.04
Grassland vs. Cropland	0.64	0.01

## Discussion

### Land use and soil chemical indicators

This study showed significant impacts of different land uses on soil quality and aggregate stability in the highlands of Northern Ethiopia. Consistent with some previous works^38,39^, our study also demonstrated the irreplaceable functions of natural forests that potentially provide ecosystem services and maintain ecosystem stability. Land use change in the Ethiopian highlands has significantly affected soil ecosystem processes, resulting in nutrient loss, soil instability and, eventually, reduced land productivity^7,9^. This study provides strong evidence for the significant decrease in SOC and TSN by 75% and 74%, respectively, in the soil of croplands and by 64% and 63% in the soils of grasslands following deforestation and subsequent land use changes. Due to the large number of parameters measured, this study focused on the 0- to 10-cm layer of the surface soil, but some studies suggest the importance of analyzing the whole soil profile to investigate the vertical trend of changes in soil attributes as soil elements may be translocated from the topsoil into the sub-surface layers in response to topsoil disturbance^40^. The significantly lower levels of SOC and TSN in the cropland compared to the natural forest were due to a rapid loss of accumulated SOC following conversion from natural forest, principally due to a lack of soil cover, an accelerated rate of soil organic matter decomposition and increased erosion^7,41^. Tillage intensity is also a major driving force of the rapid turnover of soil organic matter and the loss of soil macro-aggregates (250 µm–2000 µm), which is associated with the loss of SOC and TSN^41,42^, and soil organic carbon and soil N are negatively influenced by overgrazing due to increased erosion and reduced above-ground organic input^43,44^. However, our data showed that the grassland had significantly higher SOC and TSN than cropland, which may be connected to the high grass root biomass turnover rate, lack of tillage, and the addition of nutrients from livestock excreta in the grassland^36^.

Soil P impoverishment has been widely cited as the principal cause of declining soil fertility in the tropics^45^, and this study showed that the conversion of natural forest to conventional cropland and open grassland decreased the bicarbonate-extractable P (i.e., labile P) by 68% and 77%, respectively. This decrease is most likely related to the erosion of the organic matter-rich topsoil that occurs when the forest is removed and the subsequent decline in microbial biomass; a large percentage of the bicarbonate-extractable P is believed to be come from the soil microbial biomass^46^. The bicarbonate-extractable P fraction is considered to be one of the plant-available forms and consists of both organic P and inorganic P components that can be released by exudates from plant roots and microbial enzymes^47^. The level of hydroxide-extractable P was higher in cropland and grassland soils, and this fraction is a mixture of organic P from the soil organic matter and the P extracted from aluminum and iron oxides in the soil matrix^46^.

The lower SOC measured in the cropland and grassland may have promoted the binding of P to aluminum and iron in the soils that led to the accumulation of P in a less-available form (hydroxide-extractable P) (Fig. 1C and D)^48^. Other studies have also shown that the highly weathered soils in the tropics have a significant capacity to sorb large amounts of phosphorus thus removing it from the soil solution, which limits the availability of inorganic phosphorus for plants^45^. Thus, the continuous vegetation cover in the tropics is vitally important for phosphorus availability because it enhances the efficiency of intrasystem P cycling by improving the soil ecosystem, including the soil microbial communities^45^.

Re-establishment of vegetation cover through area exclosure and eucalyptus afforestation increased SOC by 47% and 54%, respectively, and TSN by 44% and 39%, compared to the cropland. Above- and belowground litter inputs and the protection of the soil against erosion are assumed to have contributed to the improvement of the SOC and TSN in the area exclosure and eucalyptus plantation^13,49,50^. In contrast to the findings of Mekuria and Aynekulu^49^, we found no increase in the plant-available bicarbonate-extractable P fraction in the exclosure soils compared to the cropland and grassland soils (Fig. 1C and D). Moist tropical forests are characterized by extremely efficient intrasystem P cycling and high and diverse litter inputs compared to monocultures such as eucalyptus but also compared to grassland and cropland^45^. The significantly low bicarbonate-extractable P in the soil of the area exclosure suggests that plant demand is higher than the supply, so P is a limiting factor. This finding suggests the need for an extended exclosure period improve the stocks of extractable P^43^, and the restoration of degraded land through exclosure may require longer periods of exclusion from anthropic interference to recover the soil quality closer to that of natural forest. Similarly, we did not observe a significant improvement in the available P fraction following the afforestation of degraded and marginalized communal lands with eucalyptus trees relative to cropland and grassland.

### Land use and soil aggregate stability (SAS)

**Figure 5:**
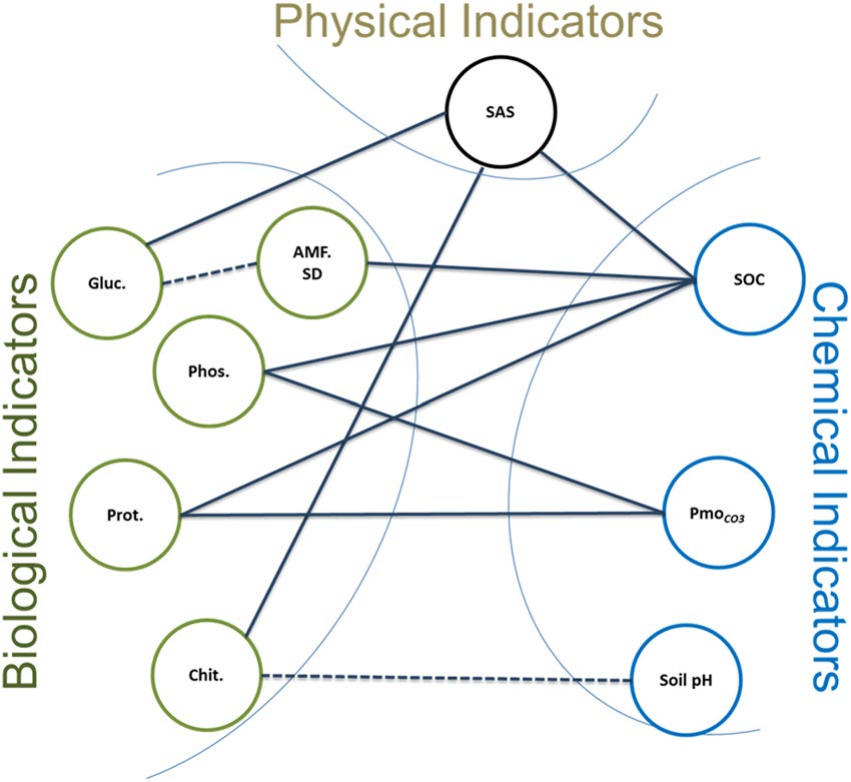
The overall correlation between the chemical, physical and biological indicators of soil quality, where AMF. SD: AM fungal spore density; Gluc: β-glucosidase; Phos: phosphatase; Prot: protease; Chit: chitinase; SAS: soil aggregate stability; SOC: soil organic carbon; Pmo_CO3_: molybdate-reactive bicarbonate-extractable P and soil pH. The total soil nitrogen strongly correlated with SOC (*ρ* = 0.93*; P* < 0.001) and similarly correlated with SAS, AMF.SD, Phos and Prot. Spearman’s correlation analysis was employed for pairwise comparisons of the three quality indicators; the solid and broken lines show significant positive and negative correlations, respectively.

Soil aggregate stability was significantly lower in cropland soils than in the soils under other land uses. Other studies have shown that tillage significantly decreases SAS^27,51^, while non-tillage promotes the stability of soil macro-aggregates (250 µm–2000 µm)^27^. Frequent tillage has been linked to a decrease in fungal hyphal biomass and a subsequent decline in the stability of soil organic matter^29,52^. In this study, soils under any form of vegetation cover had higher SAS than that measured in cropland, which shows the importance of litter inputs and a possible enhancement of soil microbial and fungal biomass with increased SAS^28,53^. The results of the ANOSIM suggested that the high SAS found in the eucalyptus soils may be due to higher microbial activity (Fig. 4). Other investigations of the effects of exclosure on soil properties have shown a slowing of the rate of organic matter decomposition^49,54^. The transition from uncontrolled grazing or degraded lands to exclosure and eucalyptus tree plantation significantly increases SAS, which may be an indicator of the recovery of SOM in these low SOM soils^24,27,52^. The linkage between SAS and the basic soil quality indicators makes SAS an easy proxy for the major factors that indicate soil quality (Fig. 5).

### Land use and AMF spore density

As discussed above, AMF fungal biomass is often strongly linked with SAS^29,55^, but our results showed no link between AMF spore density and SAS (Table 2). This may be due to the differences in sporulation and biomass production among different AMF species in response to different land uses and management^56^. Thus, AMF spore density alone is barely sufficient to provide information about mycorrhizal biomass and ultimately may not be a stand-alone indicator of soil stability. For example, the study undertaken by de Souza *et al*.^57^, revealed that the family *Gigasporaceae* has a high mycelium biomass but is characterized by low sporulation. Thus, although AMF spore density is relatively easy to measure as a soil quality indicator, it cannot be used as a proxy for the effects of fungal hyphal biomass on soil quality.

### Effects of land use change on soil enzyme activities and the overall interaction of soil quality indicators

Soil enzyme activity plays a key role in the biochemical functioning of soils, so it can be a useful biomarker for monitoring the effects of land use change on soil quality^36^. The analysis of similarity (ANOSIM) of the overall pattern of the enzyme activities showed a significant separation among the land use types, but the pairwise ANOSIM showed some overlap in enzyme activities between natural forest and eucalyptus plantation and exclosure and cropland. The results also demonstrated a significant decline in major enzyme activities in cropland. These findings are supported by previous studies^58^ of the influence of land management practices, agriculture residue management and tillage on microbial enzyme activities in response to a change in substrate supply and soil conditions. In this study, we found a significant relationship between the SAS and *β*-glucosidase and chitinase. The enzymatic activities under different land uses vary depending on the type of substrates available in the soil, which may indicate differences in SAS^59^. Factors contributing to the higher *β*-glucosidase activities in grassland may include livestock grazing and their addition of excreta, high grass root turnover and the absence of tillage^36^. This result is also consistent with the results of other researchers^33,34^, who found high enzyme activities in grassland due to the extensive accumulation of cellulosic grass root biomass that were significantly higher than in cropland.

In this study, we observed correspondence and interdependencies among chemical, physical and biological soil quality indicators, although the strengths of all correlations among them were moderate. Thus, different soil quality indicators should be taken into consideration when evaluating soil quality. Soil organic C, TSN and SAS correlate with many soils quality indicators, so they may be useful and easy-to-measure indicators for soil quality monitoring. The results of this study demonstrated that SOC is associated with soil enzymes (e.g., phosphatase and protease) that lead to the mineralization of essential nutrients (e.g., the release of plant-available P and N) and significantly correlated with TSN. Therefore, SOC is a major driver of all other soil quality indicators (Fig. 5). The correlation between the SOC, TSN and SAS is consistent with the work of Gelaw *et al*.^24^, who indicated the significant role of SOC and TSN as aggregate-binding agents in the formation of macro-aggregates. It has been suggested that the management of SOC and TSN is fundamental to the protection of the aggregate structure, which again maintains soil ecosystem functions^51^, and the formation of macro-aggregates in soils in turn enhances the lifespan of SOC and TSN accumulation by providing physical protection against decomposers^60^. In this study, we observed that cropland soils have lower SOC, TSN and SAS, and this is linked to the reduction of soil macro-aggregates because of the decline in microbial biomass. This result is corroborated by other studies^29,41,60^, which have shown significant effects of land management on mycorrhizal growth and the increased oxidation of organic carbon. Furthermore, the lower soil enzyme activities found in cropland agree with Bandick and Dick^34^ and Acosta-Martinez *et al*.^36^, who found links between the decrease in organic matter content, microbial growth and their activities, poor soil aggregate stability, and erosion. In several studies of the effects of forest soil pH gradients^61,62^, the fungal/bacterial ratio was shown to decrease with increasing soil pH, and this relationship may explain the negative correlation between soil pH and chitinase activities as the latter was significantly correlated with SAS (Fig. 5). The high chitinase activities and lower soil pH under eucalyptus soil are potentially associated with high fungal biomass production. The fungal biomass indicates a high concentration of chitin^31^ that could be the factor responsible for the high SAS in the eucalyptus plantation soil^63^, and this association enhances SOC, TSN and potential enzyme activities^24,58^.

## Conclusion

The history of uncontrolled deforestation and land degradation in the Ethiopian highlands, which transformed the natural ecosystem into an agricultural landscape, has affected the capacity of soils to provide ecosystem services. Our study shows that cropland had a significant lower organic matter and aggregate stability and thereby lowers potential soil enzyme activities than natural forest, except β-glucosidase where no statistical differences were observed. Degraded communal lands can be rehabilitated through exclosure and afforestation as a proactive way to restore the lost soil quality, particularly SAS. Our results show that a higher SAS can be achieved under all of the investigated types of vegetation cover. SAS and soil organic matter are the two parameters that first should be taken into consideration when monitoring the degradation and restoration of the soil ecosystem in response to land use change. We evaluated the most biologically active layers of the soil profile, but for a deeper understanding of the dynamics, soil quality indicators along the whole soil profile should be studied. Our results imply that the status quo of unsustainable and sedentary farming practices in the highlands of Ethiopia will have a detrimental effect on soil quality. Our finding also highlights that establishment of continuous land cover, e.g., area exclosure that support natural regeneration, and afforestation can reinforce the restoration of soil quality.

## Materials and Methods

### Study area

**Figure 6:**
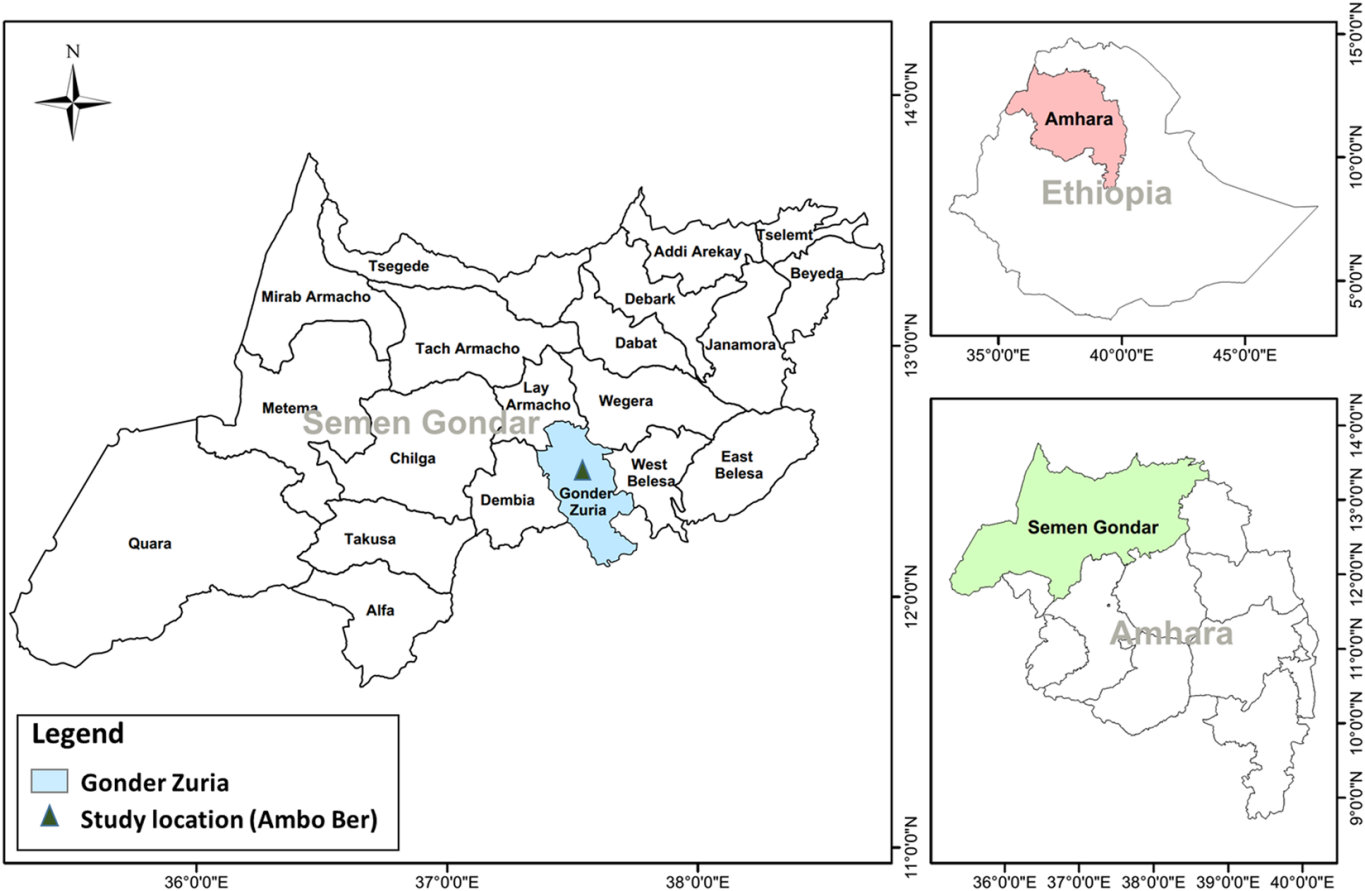
Map of Ambo Ber District, the study area. The map was produced using ESRI ArcGIS software (version 10.2; http://www.esri.com/software/arcgis/arcgis-for-desktop). The mapping data were acquired from the spatial database of the Global Administrative Areas (GADM) (Global Administrative Areas (2016). GADM database of Global Administrative Areas, version 2.8. www.gadm.org).

The study was conducted in the rural district of Ambo Ber in the North Gondar Zone of Amara Regional State, northern Ethiopia between 12°31′2.87″N and 37°31′24.37″E, approximately 30 km south of the town of Gondar (Fig. 6). Rainfall in the study area is seasonal and occurs from June to September. The mean total annual precipitation is 1,177 mm, and the mean monthly temperature varies from 18 °C in August to 22.5 °C in April. The natural forest coverage in the study area has rapidly decreased during the last 55 years at the expense of the expansion of agricultural land coupled with aggressive eucalyptus plantation^11,12^.

As described by several other researchers in the highlands of northern Ethiopia^11,12^, the natural forests in Ambo Ber were converted to the current land uses due to the high demand for agriculture and wood products, and the five land uses investigated in this study were (i) Natural forest, which is characterized by mature forest; (ii) Eucalyptus plantation, which is composed of a ca. 30-year-old *Eucalyptus camaldulensis* stand; (iii) Grassland, which is characterized by free grazing; (iv) Cropland, which is characterized by the rotational cultivation of the dominant annual crops in the area: teff (*Eragrostis tef*), wheat (*Triticum* spp.), barley (*Hordeum* spp.), maize (*Zea mays* L.) and sorghum (*Sorghum bicolor* L. Moench); and (v) Exclosure, which is degraded land that was recovered through the exclusion of human and animal interference for the last 7 years. The five land use categories are adjacent to each other and located at an elevation between 2200 m and 2300 m above sea level. The detailed site characteristics can be found online as Supplementary Table S1.

### Soil sampling

Ten composite soil samples, each composed of five sub-samples from the 0–10-cm topsoil layer were collected along two parallel transects established along the contours of each of the five land uses. The location of the first sample was randomly selected from approximately 50 m from the edge of the land use, and the following samples were taken at 50- or 100-m intervals along the transect. The second transect line was established 50 to 100 m from the first transect, depending the coverage area of the land use (see the layout in the online Supplementary Figure S1). Each of the ten composite samples were homogenized in the field using a sieve (2 mm) to remove stones, roots, macro-fauna and litter materials, and the soil samples were stored at 4 °C until analysis of the soil physicochemical parameters, soil enzyme activities and arbuscular mycorrhizal spore density (AMF-SD).

### Soil Aggregate Stability

Soil wet aggregate stability (SAS) was measured using air-dried soil. Ten replicate 4-g soil samples from each land use were subjected to wet sieving for five minutes according to the methods and apparatus of ÖNORM L 1072, 2014-04-01 (Austrian Standard). Briefly, soil particles were rinsed over a 0.25-mm sieve, and the retained soil particles were dried overnight (24 h) at 105 °C. The soil macro-aggregates (>0.25 mm in diameter) that remained after drying were weighed and subjected to dispersion in a solution of 25 cm^3^ of 0.1 M sodium pyrophosphate decahydrate (Na_4_P_2_O_7_) for 2 h to determine the sand content. The wet stable aggregate was then estimated as the mass of the aggregated soil remaining after wet sieving as a percent of the total mass of the soil without sand (equation 1).

1 $$ \% {\rm{WSA}}=(\frac{{{\rm{M}}}_{{\rm{A}}+{\rm{S}}}-{{\rm{M}}}_{{\rm{S}}}}{{{\rm{M}}}_{{\rm{T}}}-{{\rm{M}}}_{{\rm{S}}}})\ast 100$$

where % WSA is the percentage of wet stable aggregate (soil aggregate stability), M_A+S_ is the mass of wet stable aggregate plus the mass of sand (g), M_S_ is the mass of sand (g), and M_T_ is the mass of the soil sample (g).

### Soil Chemical Analyses

Soil pH was measured using a suspension of air-dried soil in deionized water at a ratio of 1:2.5 (weight/volume). Organic C and total N were analyzed with a LECO CN analyzer (TruSpec® CN, LECO Inc.), and soil phosphates were obtained by measuring the inorganic P using the modified methods described by Hedley and Stewart^64^ and Cherubin *et al*.^48^. Briefly, phosphorus was sequentially extracted from 1 g of the dry soil samples for 16 h in the following order: labile phosphorus using 30 ml 0.5 M of NaHCO_3_ and moderately labile phosphorus with 30 ml of 0.1 M NaOH. The concentration of the molybdate-reactive P fraction was determined colorimetrically from an extract of both NaHCO_3_ (molybdate-reactive bicarbonate-extractable P) and NaOH (molybdate-reactive hydroxide-extractable P)^65^ after adjusting the pH using 100 µl of 1 M HCl to 1 ml of the NaHCO_3_ and 50 µl of H_2_SO_4_ to 1 ml of the NaOH, respectively.

### Quantification of AMF spore density

Spores were isolated from soil samples by wet sieving^66^ combined with a sucrose centrifugation method^67,68^. Five grams of air-dried, sieved soil samples were mixed with 200 cm^3^ of distilled water, shaken for 15 minutes and then left to stand for 30 minutes to allow sedimentation of coarse materials. The suspension was then decanted over a metallic sieve with a mesh size of 38 μm, and the soil aggregates left on the sieve were continuously washed with distilled water until most of the soil particles were washed away. The extracted spores under the sieve were rinsed with distilled water in a separate 60-cm^3^ centrifuge tube and centrifuged at 700 *g* for 5 minutes. The floating debris was then carefully discarded, and the remaining pellet was suspended in a 50% sucrose solution and centrifuged at 700 *g* for 2 minutes. The spores found in the supernatant were poured through a 38-µm sieve and quickly washed with abundant water to remove the sucrose. The spores were rinsed with distilled water from the sieves on to a filter paper, and spore density was estimated using the direct counting method under a dissecting microscope (INVAM, http://invam.wvu.edu/).

### Soil Enzyme Activity Analyses

The assay employed in this study estimates the “maximum potential” enzymatic activity and not the actual enzyme activity^69^. The enzymes assessed in the soil samples were β-glucosidase, which mobilizes C; chitinase (N-acetyl-glucosaminidase), which mobilizes N from chitin; acid phosphatase, which mobilizes P; and protease (leucine amino peptidase), which mobilizes N. Potential enzymatic activity was measured using fluorescently labelled substrates; we used 7-amino-4-methylcoumarin (AMC) for protease and 4-methylumbelliferone (MUB) for the others^70^. The soil slurry was prepared with 0.5 g of soil in a 100-ml Erlenmeyer flask mixed with 50 ml of 100 mM 2-N-morpholino-ethanesulfonic acid (MES) buffer at pH 6.5^70,71^. The slurry was placed on a magnet stirrer for 8 min until all the larger particles were dissolved and then sonicated in a sonicator bath for 1 min at 10% power. Two hundred µl of the slurry was mixed with 50 µl of the substrate in black micro plates using three replicates per sample per enzyme, and the plates were then incubated for two hours on a plate mixer held at a constant temperature of 21 °C. The assay was measured on a fluorescence spectrophotometer (Perkin Elmer Enspire Plate Reader). A standard curve was prepared with MUB and AMC both with and without soil slurry to correct for the quenching caused by the soil particles. The potential enzyme activities were calculated in units of nmol h^−1^ g^−1^ dry mass according to Marx *et al*.^70^.

### Statistical analysis

All statistical analyses were carried out using the PAST program^72^. Statistical differences in soil aggregate stability, soil organic carbon (SOC), available soil P and total N, AMF spore density, and soil enzyme activity among the five land use categories were analyzed by one-way ANOVA. When the differences were significant, the data were further analyzed using a Tukey post hoc test (P < 0.05) to assess differences between the five land uses. All datasets were tested for normality using Jarque-Bera tests, and the equality of group variances was examined using Levene’s test. Data were square root or log transformed when necessary. Principal component analysis (PCA) was used to visualize and investigate the overall pattern of enzyme activities across land use categories and to determine the relationships between the soil enzyme activities and the land use categories. Correlations among soil quality indicators were analyzed using non-parametric Spearman’s rank correlation coefficients.

## Electronic supplementary material

Supplementary Information

## References

[1] Foley JA (2005). Global consequences of land use. science.

[2] Newbold T (2015). Global effects of land use on local terrestrial biodiversity. Nature.

[3] Elliott E (1986). Aggregate structure and carbon, nitrogen, and phosphorus in native and cultivated soils. Soil Science Society of America Journal.

[4] Islam K, Weil R (2000). Land use effects on soil quality in a tropical forest ecosystem of Bangladesh. Agriculture, Ecosystems & Environment.

[5] Bustamante M (2016). Toward an integrated monitoring framework to assess the effects of tropical forest degradation and recovery on carbon stocks and biodiversity. Global change biology.

[6] Teketay D (2001). Deforestation, wood famine, and environmental degradation in Ethiopia’s highland ecosystems: urgent need for action. Northeast African Studies.

[7] Lemenih M, Karltun E, Olsson M (2005). *A*ssessing soil chemical and physical property responses to deforestation and subsequent cultivation in smallholders farming system in Ethiopia. Agriculture, Ecosystems & Environment.

[8] Tesfaye MA, Bravo F, Ruiz-Peinado R, Pando V, Bravo-Oviedo A (2016). Impact of changes in land use, species and elevation on soil organic carbon and total nitrogen in Ethiopian Central Highlands. Geoderma.

[9] Nyssen J (2004). Human impact on the environment in the Ethiopian and Eritrean highlands—a state of the art. Earth-science reviews.

[10] Kettle WD, Rich PM, Kindscher K, Pittman GL, Fu P (2000). Land‐Use History in Ecosystem Restoration: A 40‐Year Study in the Prairie‐Forest Ecotone. Restoration Ecology.

[11] Tekle K, Hedlund L (2000). Land cover changes between 1958 and 1986 in Kalu district, southern Wello, Ethiopia. Mountain research and development.

[12] Zeleke G, Hurni H (2001). Implications of land use and land cover dynamics for mountain resource degradation in the Northwestern Ethiopian highlands. Mountain research and development.

[13] Teferi E, Bewket W, Simane B (2016). Effects of land use and land cover on selected soil quality indicators in the headwater area of the Blue Nile basin of Ethiopia. Environmental monitoring and assessment.

[14] Jaleta D, Mbilinyi B, Mahoo H, Lemenih M (2016). Eucalyptus expansion as relieving and provocative tree in Ethiopia. Journal of Agriculture and Ecology Research International.

[15] Lemenih M, Olsson M, Karltun E (2004). Comparison of soil attributes under Cupressus lusitanica and Eucalyptus saligna established on abandoned farmlands with continuously cropped farmlands and natural forest in Ethiopia. Forest ecology and management.

[16] Cossalter, C. & Pye-Smith, C. *Fast-wood forestry: myths and realities*. Vol. 1 (CIFOR, 2003).

[17] Senbeta F, Teketay D (2001). Regeneration of indigenous woody species under the canopies of tree plantations in Central Ethiopia. Tropical Ecology.

[18] Liang J, Reynolds T, Wassie A, Collins C, Wubalem A (2016). Effects of exotic Eucalyptus spp. plantations on soil properties in and around sacred natural sites in the northern Ethiopian Highlands. AIMS Agricult. Food.

[19] Karlen D (1997). Soil quality: a concept, definition, and framework for evaluation (a guest editorial). Soil Science Society of America Journal.

[20] Doran JW, Zeiss MR (2000). Soil health and sustainability: managing the biotic component of soil quality. Applied Soil Ecology.

[21] Doran JW (2002). *Soil health and global su*stainability: translating science into practice. Agriculture, ecosystems & environment.

[22] Jastrow J, Miller R, Lussenhop J (1998). Contributions of interacting biological mechanisms to soil aggregate stabilization in restored prairie. Soil Biology and Biochemistry.

[23] Wang X, Yost R, Linquist B (2001). Soil aggregate size affects phosphorus desorption from highly weathered soils and plant growth. Soil Science Society of America Journal.

[24] Gelaw AM, Singh B, Lal R (2015). Organic carbon and nitrogen associated with soil aggregates and particle sizes under different land uses in Tigray, Northern Ethiopia. Land Degradation & Development.

[25] Jastrow JD, Amonette JE, Bailey VL (2007). Mechanisms controlling soil carbon turnover and their potential application for enhancing carbon sequestration. Climatic Change.

[26] Demenois, J., Carriconde, F., Rey, F. & Stokes, A. Tropical plant communities modify soil aggregate stability along a successional vegetation gradient on a Ferralsol. *Ecological Engineering* (2017).

[27] Six J, Elliott E, Paustian K (2000). Soil macroaggregate turnover and microaggregate formation: a mechanism for C sequestration under no-tillage agriculture. Soil Biology and Biochemistry.

[28] Wu Q-S, Srivastava A, Cao M-Q, Wang J (2015). Mycorrhizal function on soil aggregate stability in root zone and root-free hyphae zone of trifoliate orange. Archives of Agronomy and Soil Science.

[29] Dai J (2015). No tillage enhances arbuscular mycorrhizal fungal population, glomalin-related soil protein content, and organic carbon accumulation in soil macroaggregates. Journal of Soils and Sediments.

[30] Zhang J, Tang X, He X, Liu J (2015). Glomalin-related soil protein responses to elevated CO 2 and nitrogen addition in a subtropical forest: Potential consequences for soil carbon accumulation. Soil Biology and Biochemistry.

[31] Bailey VL, Smith JL, Bolton H (2002). Fungal-to-bacterial ratios in soils investigated for enhanced C sequestration. Soil Biology and Biochemistry.

[32] Miller, R. & Jastrow, J. In *Arbuscular mycorrhizas: physiology and function* 3–18 (Springer, 2000).

[33] Dick, R. P. Soil enzyme activities as indicators of soil quality. *Defining soil quality for a sustainable environment*, 107–124 (1994).

[34] Bandick AK, Dick RP (1999). Field management effects on soil enzyme activities. Soil Biology and Biochemistry.

[35] Gil-Sotres F, Trasar-Cepeda C, Leirós M, Seoane S (2005). Different approaches to evaluating soil quality using biochemical properties. Soil Biology and Biochemistry.

[36] Acosta-Martinez V, Cruz L, Sotomayor-Ramirez D, Pérez-Alegría L (2007). Enzyme activities as affected by soil properties and land use in a tropical watershed. Applied Soil Ecology.

[37] Purahong, W. *et al*. Tree species, tree genotypes and tree genotypic diversity levels affect microbe-mediated soil ecosystem functions in a subtropical forest. *Scientific Reports* **6** (2016). 10.1038/srep36672 PMC5114573 27857198

[38] Kindu M, Schneider T, Teketay D, Knoke T (2016). Changes of ecosystem service values in response to land use/land cover dynamics in Munessa–Shashemene landscape of the Ethiopian highlands. Science of The Total Environment.

[39] Pan Y (2011). A large and persistent carbon sink in the world’s forests. Science.

[40] Angers D, Eriksen-Hamel N (2008). Full-inversion tillage and organic carbon distribution in soil profiles: a meta-analysis. Soil Science Society of America Journal.

[41] Cates AM, Ruark MD, Hedtcke JL, Posner JL (2016). Long-term tillage, rotation and perennialization effects on particulate and aggregate soil organic matter. Soil and Tillage Research.

[42] Liu S (2015). Effects of different tillage practices on soil water-stable aggregation and organic carbon distribution in dryland farming in Northern China. Acta Ecologica Sinica.

[43] Neff J, Reynolds R, Belnap J, Lamothe P (2005). Multi‐decadal impacts of grazing on soil physical and biogeochemical properties in southeast Utah. Ecological Applications.

[44] Steffens M, Kölbl A, Totsche KU, Kögel-Knabner I (2008). Grazing effects on soil chemical and physical properties in a semiarid steppe of Inner Mongolia (PR China). Geoderma.

[45] Oberson, A. *et al*. In *Biological Approaches to Sustainable Soil Systems* 531–546 (CRC Press, 2006).

[46] Cross AF, Schlesinger WH (1995). A literature review and evaluation of the. Hedley fractionation: Applications to the biogeochemical cycle of soil phosphorus in natural ecosystems. Geoderma.

[47] Shen J (2011). Phosphorus dynamics: from soil to plant. Plant physiology.

[48] Cherubin MR (2016). Phosphorus pools responses to land-use change for sugarcane expansion in weathered Brazilian soils. Geoderma.

[49] Mekuria W, Aynekulu E (2013). *Excl*osure land management for restoration of the soils in degraded communal grazing lands in northern Ethiopia. Land Degradation & Development.

[50] Smith P (2008). Land use change and soil organic carbon dynamics. Nutrient Cycling in Agroecosystems.

[51] Wei G (2014). Long-term effects of tillage on soil aggregates and the distribution of soil organic carbon, total nitrogen, and other nutrients in aggregates on the semi-arid loess plateau, China. Arid Land Research and Management.

[52] Du Z, Ren T, Hu C, Zhang Q (2015). Transition from intensive tillage to no-till enhances carbon sequestration in microaggregates of surface soil in the North China Plain. Soil and Tillage Research.

[53] An S, Mentler A, Mayer H, Blum WE (2010). Soil aggregation, aggregate stability, organic carbon and nitrogen in different soil aggregate fractions under forest and shrub vegetation on the Loess Plateau, China. Catena.

[54] Damene S, Tamene L, Vlek PL (2013). Performance of exclosure in restoring soil fertility: a case of Gubalafto district in North Wello Zone, northern highlands of Ethiopia. Catena.

[55] Gupta V, Germida J (1988). Distribution of microbial biomass and its activity in different soil aggregate size classes as affected by cultivation. Soil Biology and Biochemistry.

[56] Jansa J (2002). Diversity and structure of AMF communities as affected by tillage in a temperate soil. Mycorrhiza.

[57] de Souza, F. A., Dalpé, Y., Declerck, S., de la Providencia, I. E. & Séjalon-Delmas, N. In *In vitro culture of Mycorrhizas* 73–91 (Springer, 2005).

[58] Udawatta RP, Kremer RJ, Adamson BW, Anderson SH (2008). Variations in soil aggregate stability and enzyme activities in a temperate agroforestry practice. Applied soil ecology.

[59] Trasar-Cepeda C, Leirós M, Gil-Sotres F (2008). Hydrolytic enzyme activities in agricultural and forest soils. Some implications for their use as indicators of soil quality. Soil Biology and Biochemistry.

[60] Denef K, Six J, Paustian K, Merckx R (2001). Importance of macroaggregate dynamics in controlling soil carbon stabilization: short-term effects of physical disturbance induced by dry–wet cycles. Soil Biology and Biochemistry.

[61] Bååth E, Anderson T-H (2003). Comparison of soil fungal/bacterial ratios in a pH gradient using physiological and PLFA-based techniques. Soil Biology and Biochemistry.

[62] Blagodatskaya EV, Anderson T-H (1998). Interactive effects of pH and substrate quality on the fungal-to-bacterial ratio and qCO 2 of microbial communities in forest soils. Soil Biology and Biochemistry.

[63] Rillig MC (2004). Arbuscular mycorrhizae, glomalin, and soil aggregation. Canadian Journal of Soil Science.

[64] Hedley M, Stewart J (1982). Method to measure microbial phosphate in soils. Soil Biology and Biochemistry.

[65] Murphy J, Riley JP (1962). A modified single solution method for the determination of phosphate in natural waters. Analytica chimica acta.

[66] Gerdemann J, Nicolson TH (1963). Spores of mycorrhizal Endogone species extracted from soil by wet sieving and decanting. Transactions of the British Mycological society.

[67] Ianson DC, Allen MF (1986). The Effects of Soil Texture on Extraction of Vesicular-Arbuscular Mycorrhizal Fungal Spores from Arid Sites. Mycologia.

[68] Brundrett, M., Bougher, N., Dell, B., Grove, T. & Malajczuk, N. Working with Mycorrhizas in Forestry and Agriculture: Australian Centre for International Agricultural Research. *Canberra, Australia* **344** (1996).

[69] German DP (2011). Optimization of hydrolytic and oxidative enzyme methods for ecosystem studies. Soil Biology and Biochemistry.

[70] Marx M-C, Wood M, Jarvis S (2001). A microplate fluorimetric assay for the study of enzyme diversity in soils. Soil Biology and Biochemistry.

[71] Chrost R, Krambeck HJ (1986). Fluorescence correction for measurements of enzyme activity in natural waters using methylumbelliferyl-substrates. Archiv für Hydrobiologie.

[72] Hammer, Ø., Harper, D. & Ryan, P. PAST-Palaeontological statistics. www.uv.es/~pardomv/pe/2001_1/past/pastprog/past.pdf, *acessado em* 2**5**, 2009 (2001).

