# Supplementary material for: Changes in land use alter soil quality and aggregate stability in the highlands of northern Ethiopia
Source: Sci Rep. 2017 Oct 19;7:13602. doi: 10.1038/s41598-017-14128-y (PMC5648896; doi:10.1038/s41598-017-14128-y)
Supplement: Supplementary file 1 — Supplementary Information [file 41598_2017_14128_MOESM1_ESM.doc]

**Supplementary Information**

Manuscript Title

**Changes in land use alter soil quality and aggregate stability in the highlands of northern Ethiopia**

Yoseph T. Delelegn^1*^, Witoon Purahong^2^, Amila Blazevic^1^, Birru Yitaferu^3^, Tesfaye Wubet^2^, Hans Göransson^1^, Douglas L. Godbold^1^

^1^BOKU–University of Natural Resources and Life Sciences, Institute of Forest Ecology, Peter-Jordan-Straße 82, 1190 Vienna, Austria; ^2^ UFZ-Helmholtz Centre for Environmental Research, Department of Soil Ecology, Theodor-Lieser-Str.4, D-06120 Halle (Saale), Germany; ^3^ARARI–Amhara Regional Agricultural Research Institute, P. O. Box 527, Bahar Dar, Ethiopia.

^*^Correspondence and requests for materials should be addressed to Yoseph T. Delelegn ([yoseph.delelegn@boku.ac.at](mailto:yoseph.delelegn@boku.ac.at) / [yosephjet@gmail.com](mailto:yosephjet@gmail.com))

***Detailed description of the study sites***

The study was conducted in Ambo Ber Rural District in the North Gondar Zone of Amara Regional State, Northern Ethiopia, located between 12°31'2.87"N and 37°31'24.37"E, approximately 30 km south of the town of Gondar. The climate is characterized by monsoonal rainfall from June to September. Mean precipitation is highest in July (304 mm) and August (270 mm). Mean total annual precipitation is 1177 mm. Mean monthly temperature varies from 18 °C in August to 22.5 °C in April. As in other parts of the Amhara region, Ambo Ber has also undergone substantial land-use change in the last decades. In particular, crop land and grasslands have expanded in the last 55 years at the expense of natural forest and shrub land. The forest cover of the region has decreased in response of the population growth. Currently, the total forest cover of the region is about 12,884 km^2^, that is, about 8.2 % of the total land area. In this study, we selected five land uses within the Ambo Ber rural district investigate the land uses change on the soil quality and aggregate stability. Previous research survey suggests that all the experimental sites were natural forest in the recent past.

The five sites representing different land uses, (i) natural forest; (ii) eucalyptus plantation *(Eucalyptus camaldulensis)*; (iii) grassland; (iv) cropland and (v) exclosure were studied. The five land use categories are adjacent to each other, and at an elevation of between 2200 and 2300 m above sea level. For detailed site characteristics see Supplementary Table S1.

*Natural forest*

The natural forest in the study area is one of the remnant forests in the locality, which is located at an elevation of 2265 – 2300 m. a. s. l. The forest is composed of old dominant trees and shrub species including, *Olea europaea* L. subsp*. cuspidata* (Wall. ex G. Don) Ciferri, *Albizia schimperiana* Oliv., *Acacia abyssinica* Hochst. ex Benth, *Croton macrostachyus* Hochst. ex Del., *Schefflera abyssinica* (A. Rich) Harms, *Myrsine africana* L, *Grewia ferruginea* Hochst. ex A. Rich., *Apodytes dimidiata* E. Mey. ex Arn.

*Eucalyptus plantation forest*

The plantation was established with *Eucalyptus camaldulensis* in the 1980s, as part of the Gondar Fuelwood Project, to address the increasing demand of firewood by the surrounding districts including Gondar town. Within state reforestation program, about 1000 ha of communal lands in the different Kebeles of the Ambo Ber rural district were planned by eucalyptus. The eucalyptus plantation currently has an average stem density of about 1500 – 2000 stems ha^-1^, and situated adjacent to the remnant natural forest. The experimental site of the eucalyptus plantation has an area of ca. 19 ha, and is located at an elevation range of 2265 – 2300 m. a. s. l.

*Grassland*

The grassland has existed since agriculture started in the area following settlement, similarly as reported for neighboring highland ecosystem in the region. The grassland (ca. 3 ha) is situated on a slope land, and located at an elevation of 2230 – 2270 m. a. s. l. The grazing system is characterized with free grazing and no pasture management practices.

*Cropland*

The crop land was originally established in the 1930s following illegal cutting of trees, and expanded afterwards, as in neighbouring highlands. Farming practices in the district are dominantly mixed crop livestock systems, and farmers practice conventional cultivation. This cultivation system is characterized by application of agrochemicals, no use of cover-crops, frequent tillage, and no mulching and biomass transfer practices. Farming is usually rain-fed agriculture, and most farmers commonly till the farmland three to five times before sowing, similar to other Ethiopian areas. Application of chemical fertilizers by smallholder farmers was below half of the national recommended rate 100 kg diammonium phosphate (21 kg P, 18 kg N) and 100 kg urea (46 kg N) ha^-1^. Farmers usually remove agricultural residues from the farmland after harvest for animal feed and fuel. A crop rotation system is used, and the major crops grown in the area are teff (*Eragrostis tef*), wheat (*Triticum spp.*), barley (*Hordeum spp*.), fava bean (*Vicia faba* L.), maize (*Zea mays* L.), and sorghum (*Sorghum bicolor* L. Moench). The cropland studied (ca. 4 ha), is situated at an average elevation of 2260 m. a. s. l.

*Exclosure*

The exclosure was established in 2007 on degraded grassland covering, ca. 2 ha. The exclosure is located on a steep slope between the altitudinal ranges of 2243 to 2300 m. a. s. l. The exclosure was established through local communities’ participation and decision making systems. After seven years of exclusion of animals and human interference, the land has recovered with a dense growth of shrub (s) and tree (t) species. The dominant shrubs and trees rehabilitated in this parcel of land are *Acokanthera schimperi* (A.DC.) Schweinf. (s/t), *Rosa abyssinica* R. Br. ex Lindl. (s)*, Dodonaea angustifolia* L.f. (s)*, Croton macrostachyus* Hochst. ex Del. (t)*, Olea europaea* L. subsp*. cuspidata* (Wall. ex G. Don) Ciferri (t)*, Acacia abyssinica* Hochst. ex Benth (t)*, Albizia schimperiana* Oliv. (t)*, Combretum collinum* Fres. (t)*, Allophylus abyssinicus (*Hochst.) Radlk. (t) and other grass species.

**Supplementary Table S1.** Main characteristics of the five selected land use types in Ambo Ber rural district, North Gondar

| **Site characteristics** | **Cropland** | **Grassland** | **Exclosure** | **Eucalyptus plantation** | **Natural forest** |
| --- | --- | --- | --- | --- | --- |
| **Mean annual rainfall (mm)** | 1177 | 1177 | 1177 | 1177 | 1177 |
| **Mean monthly T (°C)** | 20.5 | 20.5 | 20.5 | 20.5 | 20.5 |
| **Soil pH** | 6.61 | 6.74 | 6.89 | 6.34 | 6.86 |
| **Altitude rage (m. a. s. l)** | 2260 | 2230 – 2270 | 2243 -2300 | 2265 – 2300 | 2265 – 2300 |
| **Soil type** | Leptosol | Leptosol | Leptosol | Leptosol | Leptosol |
| **Land use history** | Converted from natural forest | Converted from natural forest  and less fertile lands | Converted from over grazed and degraded lands | Converted from communal  degraded and marginal lands | Primary forest |
| **Land use age** | ≥ 40 yr | ≥ 40 yr | 7 yr | ≥ 30 yr | Old forest |
| **Dominant veg./crop** | *Eragrostis tef,*  *Hordeum spp., Vicia faba L., Zea mays L.* | *Highland perennial grass species* | *Olea europaea,*  *Albizia schimperiana,*  *Croton macrostachyus,*  *Acacia abyssinica*  *Carissa edulis,*  *Dodonaea*  *angustifolia*  *Calpurnia aurea* | *Eucalyptus camaldulensis* | *Olea europaea,*  *Albizia schimperiana,*  *Croton macrostachyus,*  *Carissa edulis,*  *Acacia abyssinica,*  *Schefflera abyssinica, Apodytes dimidiata* |

**Supplementary Table S2:** Statistical data for the soil quality indicators across the land uses in Ambo Ber Rural District.

|  | **AMF - SD (no./ g soil)** | | | | | **SAS (%)** | | | | | **Soil pH** | | | | |
| --- | --- | --- | --- | --- | --- | --- | --- | --- | --- | --- | --- | --- | --- | --- | --- |
| ***Land uses*** | **MIN** | **MAX** | **MEDIAN** | **MEAN** | **SD** | **MIN** | **MAX** | **MEDIAN** | **MEAN** | **SD** | **MIN** | **MAX** | **MEDIAN** | **MEAN** | **SD** |
| ***Cropland*** | 32,60 | 110,60 | 76,70 | 77,86 | 22,98 | 30,62 | 52,35 | 38,00 | 41,20 | 8,29 | 6,33 | 6,79 | 6,61 | 6,61 | 0,14 |
| ***Grassland*** | 12,40 | 72,80 | 40,10 | 40,64 | 23,44 | 40,01 | 75,76 | 67,93 | 64,77 | 11,57 | 6,60 | 6,89 | 6,75 | 6,74 | 0,11 |
| ***Exclosure*** | 58,20 | 190,80 | 92,70 | 102,32 | 38,29 | 49,70 | 85,36 | 75,15 | 72,03 | 13,43 | 6,41 | 7,49 | 6,88 | 6,89 | 0,29 |
| ***Eucalyptus*** | 57,00 | 99,40 | 73,90 | 76,96 | 15,20 | 71,12 | 86,52 | 84,26 | 81,56 | 5,47 | 5,66 | 6,72 | 6,41 | 6,37 | 0,31 |
| ***Natural Forest*** | 70,80 | 213,20 | 128,30 | 129,20 | 47,00 | 27,87 | 80,42 | 69,28 | 65,32 | 15,47 | 6,45 | 7,34 | 6,80 | 6,86 | 0,29 |

|  | **SOC (%)** | | | | | **TSN (%)** | | | | | **Bicarbonate Extractable P (µg/ g soil)** | | | | | **Hydroxide Extractable P (µg/ g soil)** | | | | |
| --- | --- | --- | --- | --- | --- | --- | --- | --- | --- | --- | --- | --- | --- | --- | --- | --- | --- | --- | --- | --- |
| ***Land uses*** | **MIN** | **MAX** | **MEDIAN** | **MEAN** | **SD** | **MIN** | **MAX** | **MEDIAN** | **MEAN** | **SD** | **MIN** | **MAX** | **MEDIAN** | **MEAN** | **SD** | **MIN** | **MAX** | **MEDIAN** | **MEAN** | **SD** |
| ***Cropland*** | 1,43 | 2,98 | 1,82 | 1,99 | 0,50 | 0,13 | 0,23 | 0,16 | 0,17 | 0,03 | 4,79 | 32,77 | 15,80 | 17,67 | 7,74 | 31,01 | 217,26 | 138,67 | 124,83 | 69,98 |
| ***Grassland*** | 2,02 | 4,57 | 2,94 | 2,98 | 0,80 | 0,17 | 0,37 | 0,24 | 0,24 | 0,07 | 4,66 | 24,87 | 9,46 | 12,84 | 8,18 | 27,54 | 198,77 | 55,57 | 79,04 | 62,38 |
| ***Exclosure*** | 2,15 | 4,94 | 3,66 | 3,78 | 0,91 | 0,18 | 0,36 | 0,33 | 0,30 | 0,06 | 3,37 | 15,16 | 4,79 | 6,68 | 4,35 | 18,49 | 74,54 | 27,93 | 35,48 | 18,07 |
| ***Eucalyptus*** | 1,44 | 6,00 | 4,51 | 4,37 | 1,49 | 0,14 | 0,42 | 0,28 | 0,27 | 0,08 | 2,85 | 31,74 | 22,93 | 19,55 | 10,03 | 26,96 | 55,47 | 46,22 | 43,89 | 9,61 |
| ***Natural Forest*** | 1,49 | 13,32 | 8,71 | 8,24 | 3,75 | 0,11 | 1,11 | 0,70 | 0,65 | 0,31 | 10,34 | 94,57 | 58,00 | 56,01 | 30,03 | 78,18 | 200,73 | 118,55 | 125,64 | 33,98 |

|  | **ß-glucosidase (nmol/ h/ g)** | | | | | **Chitinase (nmol/ h/ g)** | | | | | **Phosphatase (nmol/ h/ g)** | | | | | **Protease (nmol/ h/ g)** | | | | |
| --- | --- | --- | --- | --- | --- | --- | --- | --- | --- | --- | --- | --- | --- | --- | --- | --- | --- | --- | --- | --- |
| ***Land uses*** | **MIN** | **MAX** | **MEDIAN** | **MEAN** | **SD** | **MIN** | **MAX** | **MEDIAN** | **MEAN** | **SD** | **MIN** | **MAX** | **MEDIAN** | **MEAN** | **SD** | **MIN** | **MAX** | **MEDIAN** | **MEAN** | **SD** |
| ***Cropland*** | 7,98 | 48,21 | 19,51 | 24,33 | 15,41 | 91,53 | 306,50 | 138,47 | 157,76 | 61,95 | 861,32 | 2051,70 | 1149,75 | 1276,45 | 364,61 | 113,87 | 143,14 | 122,01 | 126,95 | 10,88 |
| ***Grassland*** | 152,06 | 400,37 | 249,63 | 260,28 | 83,64 | 365,22 | 834,83 | 589,79 | 595,99 | 170,07 | 1767,01 | 2522,20 | 2198,54 | 2178,34 | 253,04 | 114,58 | 202,16 | 141,79 | 143,77 | 27,14 |
| ***Exclosure*** | 37,87 | 83,11 | 63,11 | 59,86 | 15,90 | 82,96 | 271,85 | 157,20 | 174,30 | 69,15 | 1136,37 | 1771,67 | 1519,24 | 1469,17 | 219,37 | 96,09 | 152,55 | 133,39 | 127,42 | 16,88 |
| ***Eucalyptus*** | 27,22 | 86,90 | 72,78 | 63,07 | 21,32 | 519,85 | 1956,14 | 825,82 | 1050,98 | 557,41 | 1234,65 | 4146,79 | 2417,84 | 2504,63 | 942,02 | 109,19 | 804,44 | 422,95 | 424,52 | 221,61 |
| ***Natural Forest*** | 8,14 | 48,09 | 19,44 | 24,41 | 15,44 | 153,12 | 586,91 | 324,41 | 339,14 | 121,87 | 1519,52 | 3899,86 | 3005,02 | 2792,87 | 803,44 | 132,97 | 559,60 | 378,06 | 367,71 | 140,43 |

*
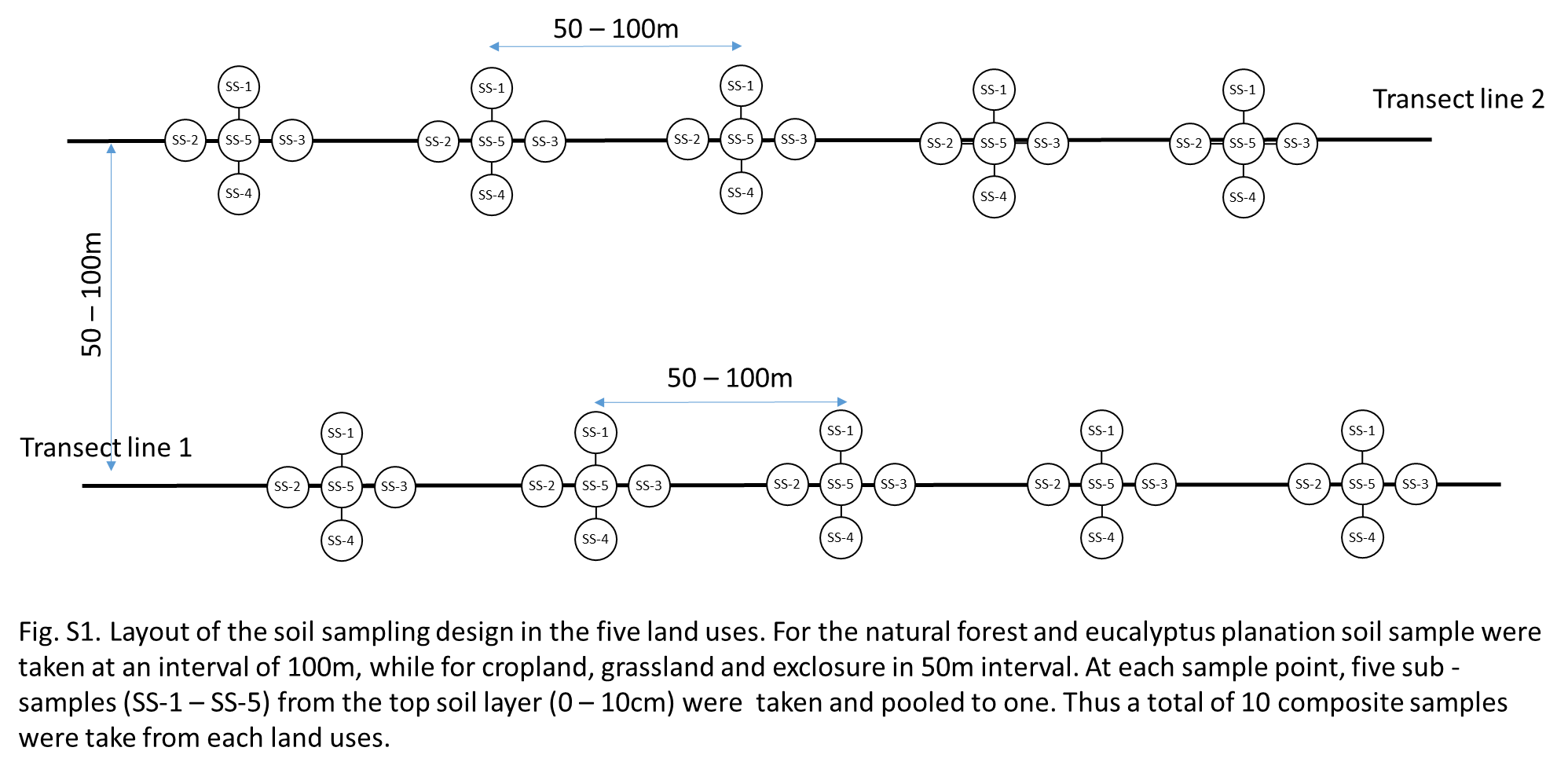
*

**Figure S1:** Layout of the soil sampling design in the five land uses. For the natural forest and eucalyptus planation soil sample were taken at an interval of 100 m, while for cropland, grassland and exclosure in 50 m interval. At each sample point, five sub - samples (SS-1 – SS-5) from the top soil layer (0 – 10cm) were taken and pooled to one. Thus a total of 10 composite samples were collected from each land uses.


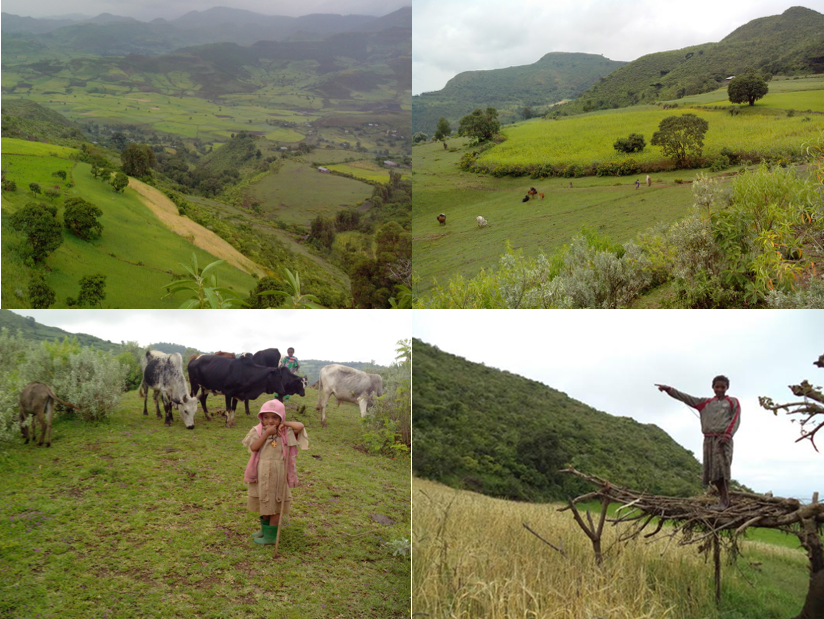


NF

EP

CL

GL

EX

EX

GL

**Figure S2**: The land use types in Ambo Ber Rural District, North Gondar. NP: Natural Forest; EP: Eucalyptus planation; CL: Cropland; GL: Grassland; EX: Exclosure (Photo by Yoseph T. D.).

***Historical Land Use Land Cover Changes in Ambo Ber Rural District, North Gondar***

The land uses land cover change in the last 40 years, showed the conversion of marginalized farmlands, grasslands, shrub lands and deforested areas to eucalyptus planation. The significant increment in the forest and woodland from the year 1972 to 1986 is most probably attributed to the aggressive afforestation program that was aimed to address the huge fuel-wood construction wood demand of urban and peri-urban communities which was strongly supported by the Derg regime. On the other hand, the decline shown between 1986 and 1999 was driven due to illegal deforestation following the fall of the Derg regime. The increase in the forest area shown in 2011 could be due to the recent eucalyptus plantation activities undertaken by the community as sources of income, and the local administration to addressing the growing demand for wood products. The decrease in farmland may be associated with decline of land productivity where local communities took eucalyptus planation as an alternative for improving livelihood.


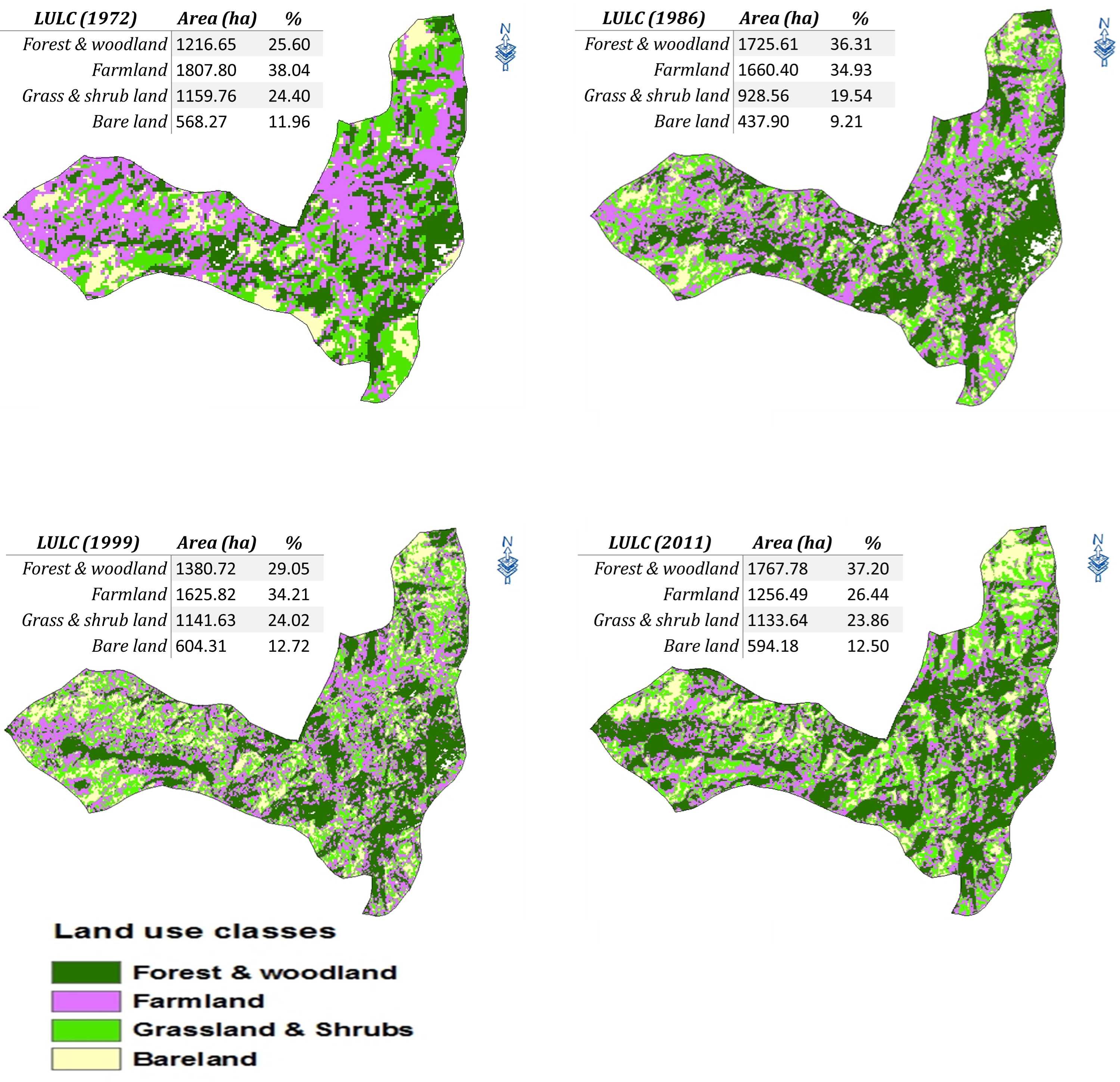


**Figure S3:** The land use map of Ambo Ber Rural District, North Gondar in 1972, 1988, 1999 and 2011. The map was generated from Landsat MSS and TM, (NASA Landsat Program, 2016, Landsat MSS scene LM01_170051_19721209, L1GS; and TM scene LT05_ 170051_2011102, L1TP; LT05_170051_19991115, L1TP; LT05_ 170051_19860103, L1GS, USGS, Sioux Falls, 1972, 1986, 1999, 2011). The map was constructed using ArcGIS software [10.3], (<http://www.esri.com/software/arcgis>).
